# Highly adaptable smartphone-based monitoring for patients with severe mental illness: Feasibility and usability study

**DOI:** 10.1177/20552076251393283

**Published:** 2025-11-13

**Authors:** Felix Machleid, Anette Schönewald, Esther Quinlivan, Linda Kokwaro, Louisa Schröder-Frekes, Toni Muffel, Caspar Wiegmann, Jakob Kaminski

**Affiliations:** 1 Department of Psychiatry and Neurosciences, Charité Campus Mitte, 14903 Charité – Universitätsmedizin Berlin, Germany; 2 Berlin Institute of Health at Charité—Universitätsmedizin Berlin, Germany; 3 27689 Vivantes Klinikum am Urban, Berlin, Germany; 4 743742 Recovery Cat GmbH, Berlin, Germany; 5 Theodor-Wenzel-Werk, Berlin, Germany

**Keywords:** Digital mental health, mobile health technologies, mHealth, remote measurement-based care, ecological momentary assessment

## Abstract

**Objective:**

Severe mental illness (SMI) requires continuous treatment. Smartphone-based monitoring enables real-time data collection and offers a way of complementing clinical workflows by creating a bridge between patients and providers. Since their needs often differ, collaborative and highly adaptable tools are warranted. The objective of this study was to evaluate the feasibility of the highly adaptable remote-measurement-based care intervention *Recovery Cat* for patients with SMI. Specifically, we aimed to assess patient adherence and retention rates of the system as well as patients’ and providers’ perceptions of its usability.

**Methods:**

Forty-nine patients were recruited for the 90-day trial and were provided with Recovery Cat a smartphone app designed to collaboratively set up and monitor clinical symptoms and functional parameters. Data were collected through daily self-reports. Feasibility was assessed by the dropout rate and user engagement, while user-friendliness was evaluated by the System Usability Scale (SUS).

**Results:**

29 participants completed the study. The majority were single, unemployed, diagnosed with an affective disorder, had prior psychiatric treatment, and were in continuous outpatient psychiatric care. EMA data were available for 26 participants. 84.61% (*n* = 22) had an adherence rate of over 50%. The mean number of days between data entry was 1.48 (sd = 2.02). The SUS indicated a positive user experience with a mean of 82.40 (sd = 11.57).

**Conclusion:**

The results suggest that highly adaptable smartphone-based monitoring is feasible for patients with SMI. High adherence rates and positive usability scores indicate that this approach holds promise for enhancing mental health care.

## Introduction

Mental illnesses have a high prevalence, leading to significant impairments on an individual and societal level^1^ and high treatment costs.^2^ Particularly, severe mental illnesses (SMIs), such as bipolar disorder, schizophrenia, and major depressive disorder, are characterized by relapsing and remitting or chronic trajectories, and frequent hospitalization.^3^ An increasing prevalence of mental disorders, especially in juveniles and young adults, has been observed in recent years.^4,5^

Patient-reported outcome measures (PROMs) for evaluating treatment response from a patient-centered perspective are increasingly used in medicine and mental health.^6^ The systematic and continuous use of PROMs to assess treatment outcomes is referred to as measurement-based care (MBC),^7^ and in the case of remote assessment in between treatment appointments, as remote MBC (RMBC). MBC encompasses two aspects: the routine assessments of outcomes, such as symptom severity, and the use of evaluations in clinical decision-making.^8^ MBC is included in clinical guidelines and ameliorates patient engagement,^6–8^ shared decision-making,^8,9^ therapeutic alliance, and treatment outcomes.^9,10^ MBC represents a potentially cost-effective and low-risk treatment component of mental illness, which seems particularly effective when combined with other established treatments like psychiatric counseling, pharmacotherapy, or psychotherapy.^11,12^

Despite the increasing use of in medical and psychotherapy research,^13^ MBC is rarely implemented in routine care.^14^ This might be due to a variety of barriers impeding the access of SMI patients to digital tools in general and, more specifically, MBC interventions. Some may not have high-speed internet access^15^ or own a smartphone.^16^ Others may lack digital literacy^17^ or have concerns about privacy and data security.^18^ Patients with psychotic disorders^19,20^ and bipolar disorder^21^ were found to have lower mean adherence rates in ambulatory assessment studies compared to healthy controls.^20^ There are also barriers on the practitioner side. For instance, MBC systems must integrate seamlessly into the treatment processes of medical practices without requiring additional time or personnel resources.

Recent hopes for individualized, personalized, and user-friendly monitoring have been connected to computerized adaptive testing of PROMs.^22^ This method substantially increases the relevance of items to the patients, while reducing the burden of the monitoring and enhancing engagement.^23^ However, this approach lacks the value of collaboratively deciding on key symptoms and features and creating common goals in treatment, a key to shared decision-making.

Based on a rigorous needs assessment and user-centered design thinking approach, including mental health practitioners, specialists, and patients, we developed a highly adaptable MBC platform^24^ with few implementation barriers for use in any outpatient psychiatric care setting. We aimed to capture accurate information on symptom trajectories, side effects of medication, quality of life, and everyday functioning through customizable electronic patient-reported outcomes (ePROs). According to the principles of user-centered design the following development steps were carried out: (1) Interviews with patients, physicians, and digital health experts to identify needs, requirements, and potential barriers to the use of the MBC tool, (2) the conception, design, iterative testing, and adaption of the prototype, which resulted in a minimal viable product (MVP)—the progressive web application *Recovery Cat* for customized symptom monitoring. The current study aimed to evaluate the feasibility and usability of the MVP Recovery Cat among patients with SMI and their practitioners in outpatient psychiatric care.

## Objectives

The current study aimed to test Recovery Cat in outpatient treatment for:

1. User engagement with the RMBC intervention
2. Perceived usability of the application.

## Methods

This case-control feasibility study was approved by the local ethics committee at Charité Universitätsmedizin Berlin (No. EA1/314/21) and preregistered (https://osf.io/hgkjt). There were no deviations from the study protocol during the conduct of this study. Before the start of the study, written informed consent was obtained from all participants. As this was a feasibility study that did not require the estimation of effect sizes, a formal sample size calculation was not performed.

### The RMBC intervention “Recovery Cat”

**Figure 1.**
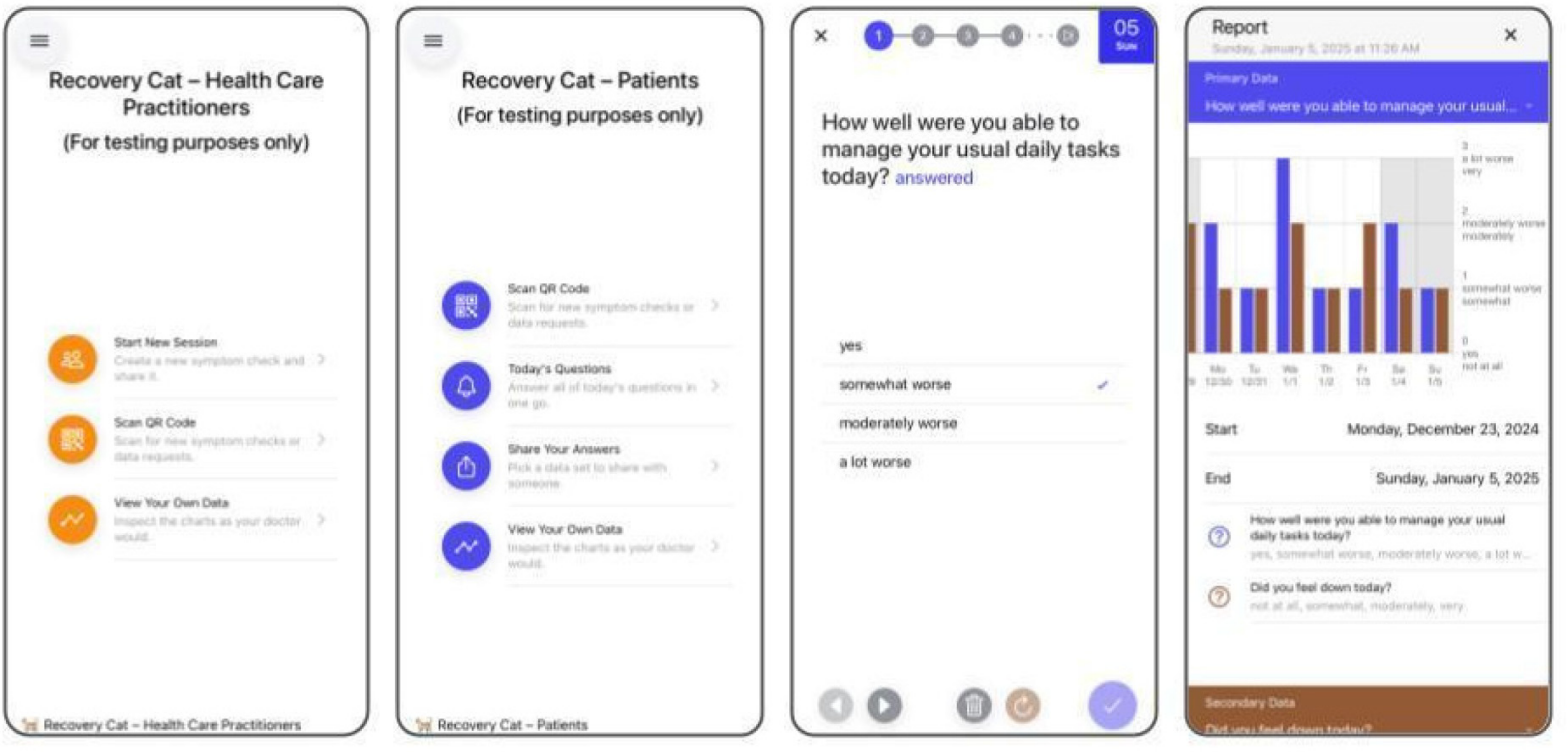
Interfaces of the MVP Recovery Cat. In the first screen, the home screen for practitioners is displayed, offering options to draft ePRO items, access patient data, and view patient information. The second screen shows the patients’ home screen, where users can receive ePRO items, view their respective data, share information with their practitioner, and access their records. The third screenshot presents an example of an ePRO item. The fourth screenshot displays patient data over one week as bar graphs, comparing two ePRO items.

MVP: minimal viable product; ePRO: electronic patient-reported outcome.

**Figure 2.**
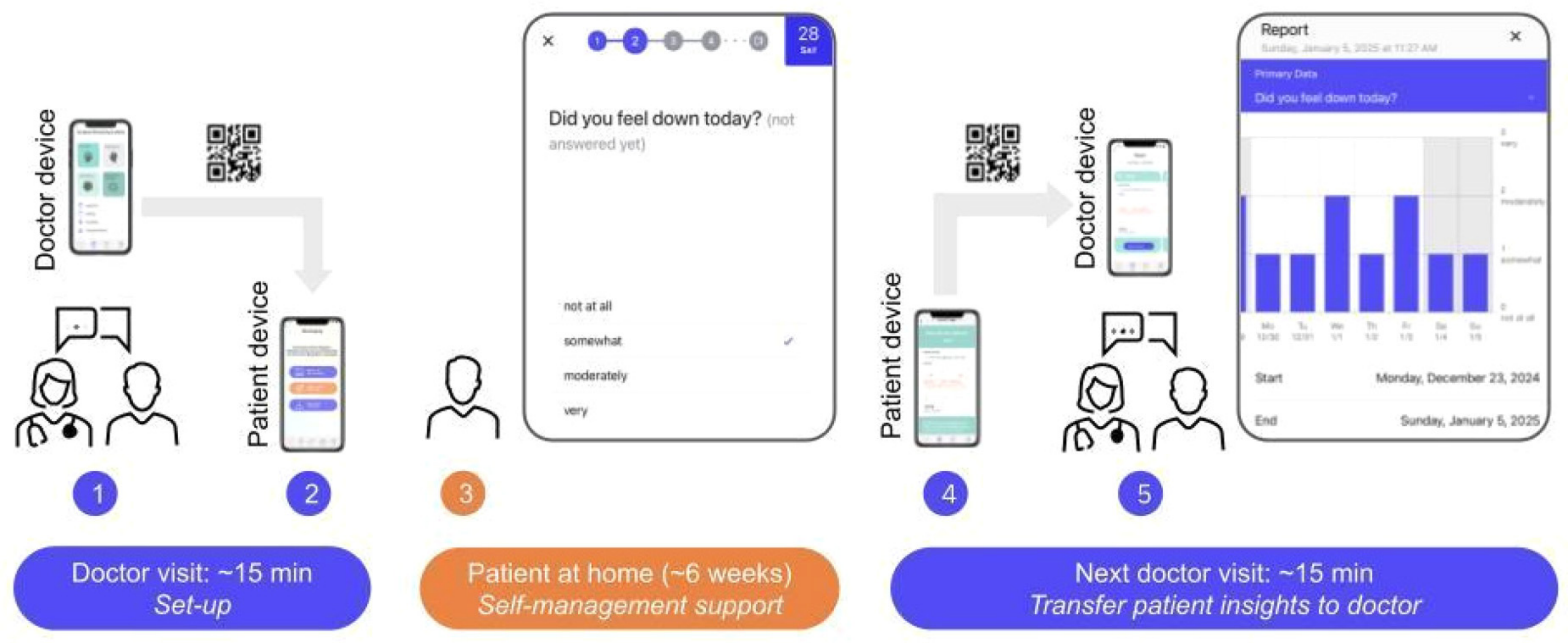
Integration of Recovery Cat into the psychiatric care journey with minimal time efforts, a safe and easy way to use data sharing mechanism (1–5) via QR-code scan, an example screen of an ePRO item (3) and data visualization (5).

Tailoring Recovery Cat to patient needs resulted in a setup with two user interfaces (Figure 1). Patients were asked to install Recovery Cat on their smartphones. Practitioners used the web application on their devices. (1) Initially, practitioners and patients configured the monitoring process using predefined ePRO items that were collaboratively developed with psychologists, psychiatrists, and patients on clinical key symptoms and functional parameters according to the respective diagnosis on the practitioner interface (Table 1, Figure 2). Additionally, patients and practitioners could collaboratively create individual ePRO items by changing the text or creating individual continuous scales, interval scales, or multiple-choice options. (2) The customized monitoring was then transferred to the patient device. (3) Patients engaged in their daily self-monitoring recording ePROs for 90 days. If entries were not completed, patients received push notifications as reminders. The study staff did not make any phone contact to prompt participation. (4) During outpatient appointments, patient data could be transferred back to the practitioners’ devices via a QR code. (5) Practitioners were encouraged to review the longitudinal ePRO data, discuss trends and the treatment process based on the visualization of the ePROs, and plan for the next steps in treatment while adapting monitoring by modifying, adding, or deleting the individualized ePROs at each appointment. To protect data privacy, ePROs and query intervals were securely transmitted via encrypted real-time communication (WebRTC protocol) without temporary storage on a central server. The QR code contained the key and connection data for transfer from the patient's smartphone to the practitioner's device. Patient answers were stored locally with password protection and could be retransmitted via WebRTC at the next outpatient appointment. The data remained visible only during the appointment and were not stored on the practitioner's device.

ePRO: electronic patient-reported outcome.

**Table 1.** ePRO items implemented for different diagnostic groups.

Domain	ePRO item	Response	ICD-10 diagnosis
Daily functioning	How well were you able to manage your usual daily tasks today?	Yes, somewhat worse, moderately worse, a lot worse	F20/F25/F31/F33
Depressed mood	Did you feel down today?	Not at all, somewhat, moderately, very	F20/F25/F31/F33
Lack of drive	Was your energy low today?	Not at all, somewhat, moderately, very	F20/F25/F31/F33
Increased drive	Were you full of energy and motivated to do lots of different things today?	Not at all, somewhat, moderately, very	F25/F31
Racing thoughts	Did you have the impression that thoughts were racing through your head today?	Not at all, rarely, often, constantly	F25/F31
Delusional mood	Did you feel like something strange was going on today?	Not at all, somewhat, moderately, a lot	F20/F25
Suspicion, Paranoia	Did you feel suspicious today?	Not at all, somewhat, moderately, very	F20/F25

ePRO: electronic patient-reported outcome.

## Participants

The study sample was recruited at the psychiatric outpatient clinics at Kliniken im Theodor-Wenzel-Werk and the Department of Psychiatry and Psychotherapy at Charité Campus Mitte in Berlin between March 2022 and May 2024. Participants had to be undergoing treatment in one of the respective psychiatric outpatient clinics, be 18 years or older, and meet the diagnostic criteria of one of the following SMI disorders: paranoid schizophrenia (ICD-10: F20), schizoaffective disorder (ICD-10: F25), bipolar affective disorder (ICD-10: F31), depressive disorder (ICD-10: F32), or recurrent depressive disorder (ICD-10: F33). Patients with acute suicidality, organic brain disease (ICD-10: F0x), substance use disorder (ICD-10: F1x), and intellectual disability (ICD-10: F7x) were excluded. Participants had to have a smartphone. While the main focus of the study was on patients, usability was also assessed among the respective treating practitioners in the psychiatric outpatient clinic.

## Data collection procedures

Immediately following study inclusion (T0), basic clinical documentation, diagnosis-unspecific, and diagnosis-specific psychometric data were collected. Participants attended outpatient visits according to their usual intervals (3–6 weeks) but additionally used Recovery Cat as described above. At the end of the 90-day intervention (T1), the baseline clinical documentation and psychometric data were reassessed. Additionally, participants and practitioners evaluated the usability of Recovery Cat using the System Usability Scale (SUS). The rate of completers, dropouts, emergency department visits, and inpatient admissions was recorded. All data were collected using REDcap software,^25,26^ exported as CSV, and stored on the Charité server.

## Definition of completers and dropouts

Participants were defined as completers if they responded to the baseline (T0) and final (T1) assessments and had ePRO data available. Additionally, participants who completed both assessments but lacked ePRO data were also considered completers if the reason for not using Recovery Cat was known (e.g. lack of motivation). Dropouts were defined as participants who provided written informed consent and completed the baseline assessment (T0) but did not complete the final assessment (T1). Participants were also classified as dropouts if they completed both the baseline and final assessment but lacked ePRO data without any information explaining nonuse, or if they informed study staff of their decision to withdraw from the study.

## Operationalization of feasibility through user engagement and user-friendliness

**Figure 3.**
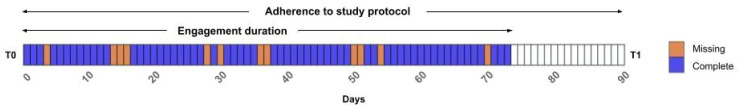
Visualization of the adherence to the study protocol and engagement duration metrics of one participant. Purple fields mark days on which at least one ePRO question was answered, while orange-colored fields indicate days without data entries.

To address the central research question, whether the smartphone-based recording of individual ePROs between outpatient appointments is feasible, three outcome parameters were defined:

1. Dropout rate: It was hypothesized that the dropout rate would remain below 60%^27^
2. User engagement: It was hypothesized that the app's usability would motivate participants to maintain continuous data entry throughout the study period. Engagement (Figure 3) was measured by
  - The adherence to the study protocol, defined as the number of days on which at least one ePRO item was returned compared to the study period of 90 days. It was hypothesized that participants would complete at least 75% of the planned ePRO days.
  - The engagement duration, defined as the adherence during the time to the final response (capped at 90 days). Engagement duration was calculated as the proportion of days participants entered ePRO data to the total number of days they used the app, regardless of the 90-day study period.
3. User-friendliness: It was hypothesized that participants and practitioners would positively evaluate the digital recording of individual ePROs using the standardized instrument of the SUS.^28^ The scale comprises 10 questions that users answer after using a system, covering different aspects such as ease of use, complexity, perceived self-confidence and competence when using the system, and the integration of system functionalities.^29^

ePRO: electronic patient-reported outcome.

## Data analysis

All statistical analyses were conducted using the statistical software RStudio (Version 2023.09.1 + 494).^30^ Basic demographic characteristics were summarized using means and standard deviations.^30^ For the analysis of medications, the total number of drugs within each medication group was calculated by summing all preparations used in the study population. This approach focused on the medications themselves rather than the number of patients, as some individuals may take multiple drugs from the same medication group. Exploratory analyses were performed to generate potential initial hypotheses for a future study. T-tests were used to test between-group differences (e.g. diagnosis) for engagement outcomes. ANOVA tests were used to test for the association between categorically defined demographic variables (e.g. income, degree) and engagement outcomes. Pearson's product-moment correlation was used to test associations between continuous variables (e.g. age) and respective outcomes. Due to the small sample size, Hedge's g was used to calculate the effect size. Results were considered statistically significant at *p* < .05. A scatter plot was used to visualize the relationship between the number of ePRO items and adherence. Pearson's correlation coefficients (*r*) were calculated to quantify the strength of association. Fisher's z-test was applied to compare the two tests to determine whether the difference between the associations was statistically significant. As this was a feasibility study, no data imputation was performed. Missing data in psychometric scales were handled using listwise deletion. Missing EMA data were considered in the engagement analysis unless the participant was classified as a dropout.

## Results

### Participant characteristics

A total of 49 participants were included in the study, of which 20 were classified as dropouts due to lack of motivation (*n* = 3), technical difficulties such as faulty display of ePRO items or accidental clearing of data (*n* = 4), relapse (*n* = 1), study cancelation (*n* = 1), and unknown reasons (*n* = 11). 29 participants were included for analysis (Table 2, Supplemental Table S1). The dropout rate for the study was 40.82%.

**Table 2.** Demographics and baseline characteristics of all participants, completers, and dropouts.

	All partici-pants (*n*)	Mean (sd)	Comple-ters (*n*)	Mean (sd)
Total	49		29	
Age		43.36 (9.66)		42.55 (9.48)
Gender				
Male	28		19	
Female	19		9	
Diverse	2		1	
Diagnosis				
F20: Paranoid schizophrenia	8		5	
F25: Schizoaffective disorder	4		2	
F31: Bipolar disorder	27		13	
F32/33: (Recurrent) depressive disorder	10		9	
Inpatient and daycare psychiatric care				
Past inpatient or daycare psychiatric treatment	42		25	
Age at first inpatient or daycare psychiatric treatment		28.54 (9.12)		27.46 (8.51)
Outpatient psychiatric care				
No information	1		0	
No consultation	1		0	
Consultation or short treatment phase	1		1	
Continuous treatment for six months or several short episodes	6		2	
Continuous treatment over years or numerous short episodes	40		25	
Psychotherapy				
Currently in psychotherapy	30		18	
Medication (total drug counts)				
Total	83		46	
Antipsychotic	37		19	
Antidepressant	22		14	
Anticonvulsant	11		4	
Anxiolytic	3		3	
Lithium	10		6	
Baseline characteristics (range)				
WST (raw values 0–42)		33.82 (4.27)		32.88 (5.19)
WHOQOL-Bref (0–100)		85.25 (13.81)		83.86 (13.82)
ReQoL (0–80)		47.17 (16.59)		45.71 (15.86)
GAF (0–100)		65.75 (15.57)		64.93 (14.14)
CGI (0–7)		3.40 (1.33)		3.46 (1.35)
MARS (0–10)		7.57 (1.74)		7.30 (1.79)
PANSS (30–210)		49.11 (14.03)		48.00 (13.75)
ESI (0–120)		22.43 (14.21)		26.13 (14.81)
EPAS (1–5)		3.50 (0.58)		3.40 (0.50)
BDI (0–63)		15.35 (9.86)		16.20 (9.92)
HAMD (0–52)		10.90 (7.24)		12.16 (6.91)
YMRS (0–60)		3.77 (6.78)		4.20 (7.46)
ASRM (0–20)		2.68 (3.94)		2.19 (3.31)

sd: standard deviation; WST: Wortschatztest Vocabulary Test; WHOQOL-BREF: World Health Organization Quality of Life-BREF; ReQoL: Recovering Quality of Life; GAF: Global Assessment of Functioning; CGI: Clinical Global Impression; MARS: Medication Adherence Rating Scale; BDI: Beck Depression; PANSS: Positive and Negative Syndrome Scale; ESI: Eppendorf Schizophrenia Inventory; EPAS: Empowerment im Prozess der psychiatrischen Behandlung von Patienten mit affektiven und schizophrenen Störungen (empowerment in the process of psychiatric treatment of patients with affective and schizophrenic disorders); HAMD: Hamilton Depression Rating Scale; YMRS: Young Mania Rating Scale; ASRM: Altman Self-Rating Mania Scale.

The average age of the participants who completed the study (*n* = 29) was 42.55 years (sd = 9.48). They were slightly younger than the control group (44.55 years, sd = 10.03), and showed an equal distribution of male and female participants. Most participants were single (*n* = 18), lived in an apartment or house (*n* = 28), and had attained a higher education level (*n* = 22). Employment status varied, with most participants being unemployed (*n* = 15), some working part-time (*n* = 8), and a few employed full-time (*n* = 6). Regarding income, two main groups emerged: one earning above €3000 (*n* = 10) and one earning below €1000 (*n* = 9). The majority of participants were diagnosed with an affective disorder (*n* = 21), primarily bipolar disorder (*n* = 13). Seven participants were diagnosed with schizophrenia. Nearly all participants (*n* = 25) had previously received inpatient or daycare psychiatric treatment, with an average age of 27.46 years (sd = 8.51) at the time of their first treatment. Likewise, almost all (*n* = 25) were in continuous outpatient psychiatric treatment or numerous short-term outpatient treatment episodes. Nineteen participants were undergoing psychotherapeutic treatment. In total, 46 psychopharmacological agents were used, with antipsychotics (*n* = 19) and antidepressants (*n* = 14) being the most common. On average, participants used 1.77 medications (sd = 1.33).

Participants who completed the study reported an above-threshold quality of life as measured by the WHOQOL-BREF (83.86, sd = 13.82) and ReQoL (45.7, sd = 15.86). They presented with mild to moderate symptoms (CGI: 3.46, sd = 1.35) and moderate challenges in social, occupational, or academic functioning (GAF: 64.93, sd = 14.14). Participants experienced an above-average level of empowerment in various areas of life (EPAS: 3.40, sd = 0.50). Regarding diagnosis-specific symptoms, respective subgroups showed mild psychotic symptoms (PANSS: 48.00, sd = 13.75; ESI: 26.13, sd = 14.81), mild depressive symptoms (BDI: 16.20, sd = 9.92, HAMD: 12.16, sd = 6.01), and minimal manic symptoms (YMRS: 4.20, sd = 7.46; ASRM: 2.19, sd = 3.31). Medication adherence was reported to be moderate (MARS: 7.30, sd = 1.79).

### User engagement

**Figure 4.**
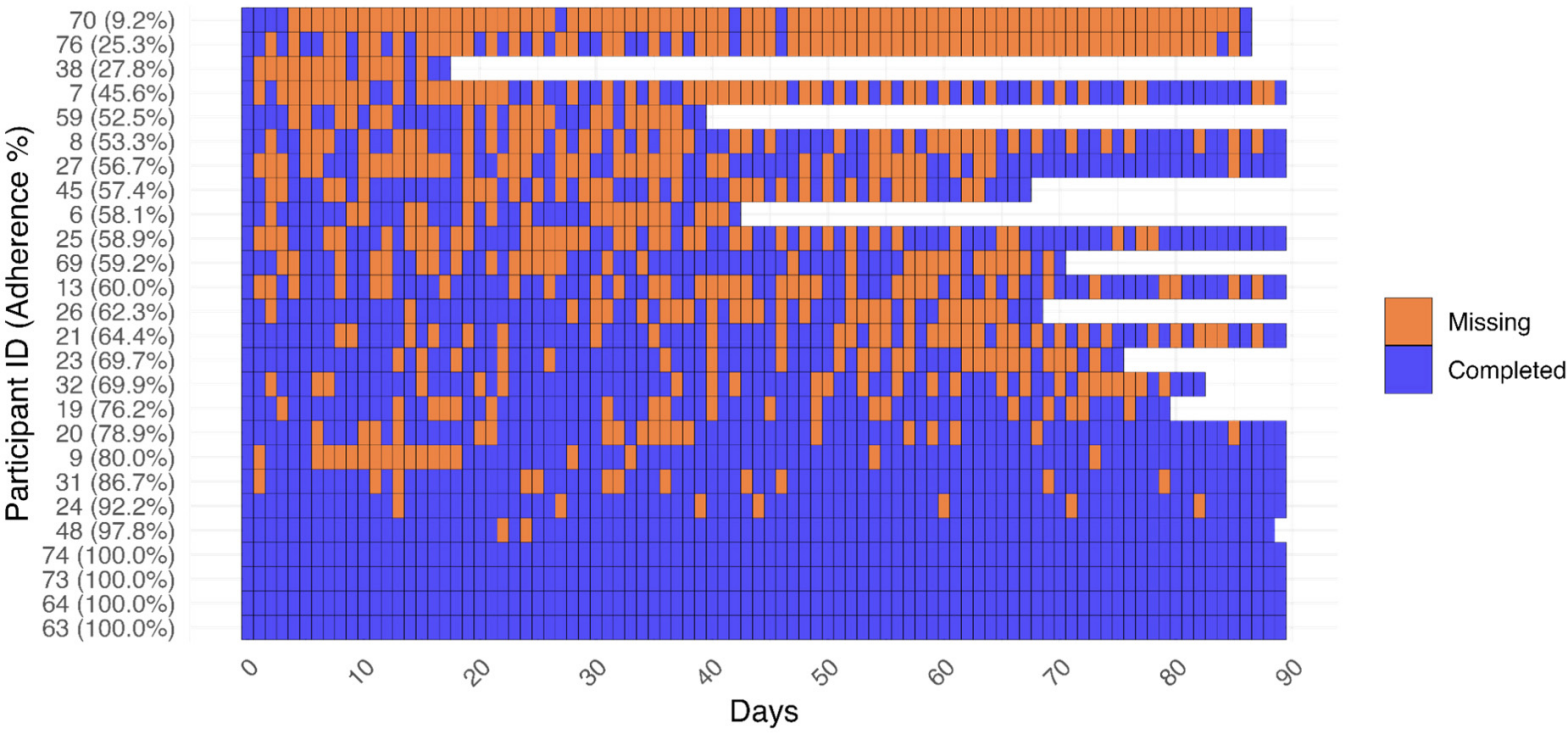
Engagement with Recovery Cat. Purple fields mark days on which at least one ePRO question was answered, while orange-colored fields indicate days without data entries.

EMA data were available for 26 of the 29 participants who completed the study. The mean amount of days between data entry was 1.48 (sd = 2.02, Supplemental Table S5) Considering engagement duration (Figure 4, Supplemental Table S3), participants, on average, entered ePRO data on 67.00% (sd = 24.25) of the days until the final response, which corresponds to a mean of 53.37 days (sd = 23.03). The engagement duration was the shortest for participant 38 (18 days), while 14 participants used the app until the end of the study (90 days). In total, 38.46% of the participants (*n* = 10) had an adherence rate of over 75%, 84.61% (*n* = 22) had an adherence rate of over 50%, and 96.15% (*n* = 25) had an adherence rate of over 25% during their participation. Adherence to the study protocol was 61.24% (sd = 28.57), corresponding to a mean of 55.5 days (sd = 25.71) during which participants used the app within the study period.

ePRO: electronic patient-reported outcome.

The association between demographic characteristics and engagement duration was tested in a subgroup analysis (Supplemental Table S4). Adherence during engagement varied by diagnostic category, with higher adherence among participants with affective disorders (71.37%, sd = 25.80) compared to those with schizophrenia and schizoaffective disorder disorders (55.13%, sd = 15.10, Supplemental Table S3) however, the difference was not statistically significant (t = 1.975, df = 18.648, *p* = .0632). Further, no significant associations were found between engagement duration and age (*r* = 1.402, df = 24, *p* = .174), gender, *F*(2,23) = 0.015, *p* = .985, income, *F*(3,21) = 0.547, *p* = .655 and severity of illness (*t* = −0.177, df = 23, *p* = .861).

**Figure 5.**
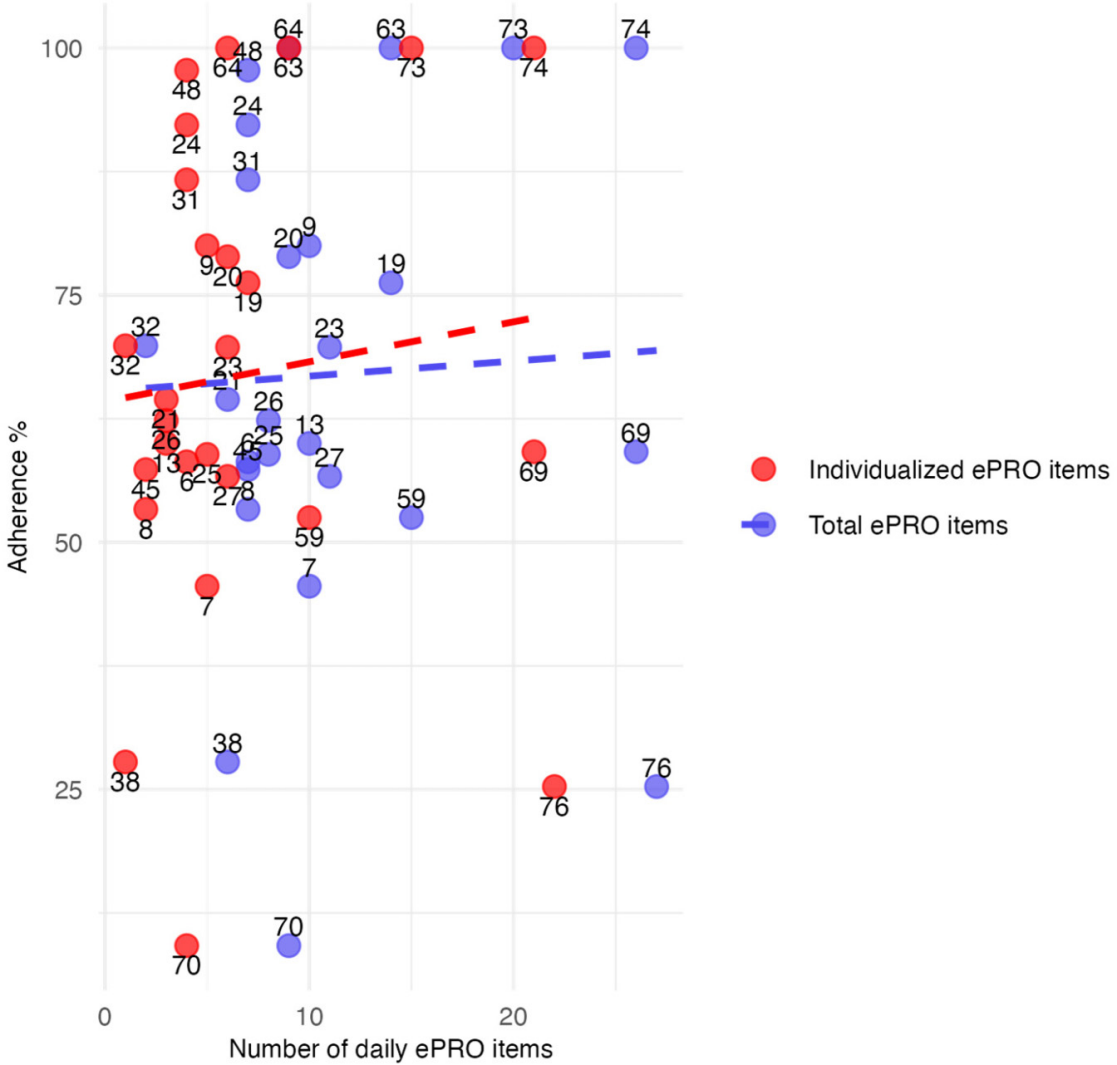
Relationship between the total number (purple) and individual daily ePRO items (red) and adherence of the study participants expressed by individual data points (with respective participant IDs) and by trend lines.

The scatter plot highlights individual differences in adherence based on the number of total and individualized daily ePRO items (Figure 5). The trend lines suggest that participants who completed more ePRO items—both in total and individualized—tended to have higher adherence. The correlation between total ePRO items and adherence was weak, at *r* = .041 (*p* = .841, purple line), while the correlation for individualized ePRO items was *r* = .101 (*p* = .622, red line). Statistical comparison using Fisher's z-test revealed no significant difference (z = −0.205, *p* = .838).

ePRO: electronic patient-reported outcome.

### User-friendliness

**Figure 6.**
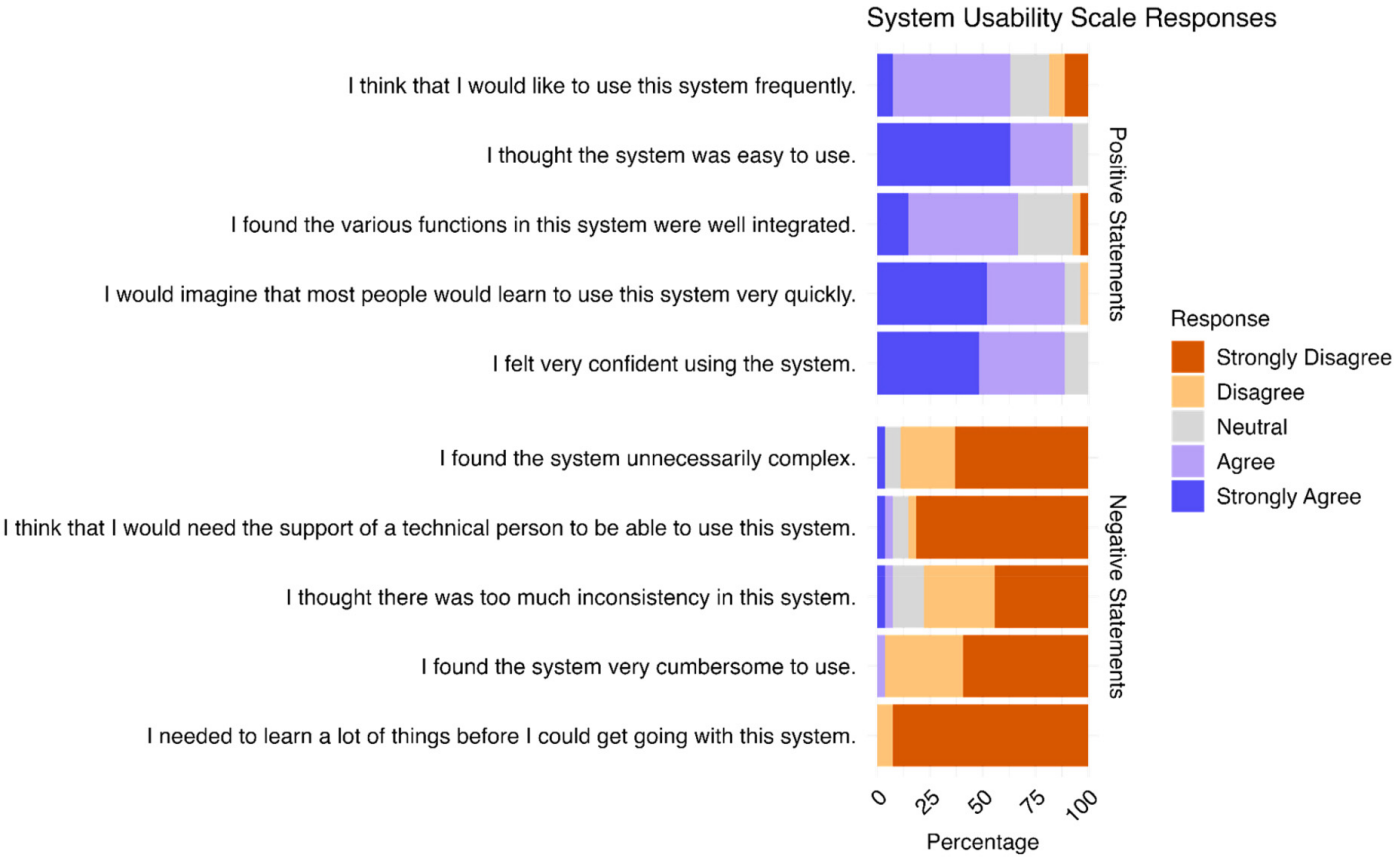
SUS of participants (*n* = 26).

Data on user-friendliness were available for 26 out of the 29 participants (see Figure 6, Supplemental Table S4). Overall, user-friendliness was rated highly (SUS scores > 70 indicate good usability^31^), with a mean score of 82.40 (sd = 11.57). Analysis of individual SUS items revealed that the majority of users expressed a preference for frequent use of Recovery Cat (74.07%) and found the app easy to use (92.58%) with well-integrated functions (66.76%). Most participants felt confident while using the app (88.89%) and did not perceive it as unnecessarily complex (88.89%), inconsistent (77.77%), or cumbersome to use (96.30%). Additionally, the majority indicated that they would not require technical support to operate Recovery Cat (85.18%) and felt no need for prior learning before its use (100%).

SUS: System Usability Scale.

**Figure 7.**
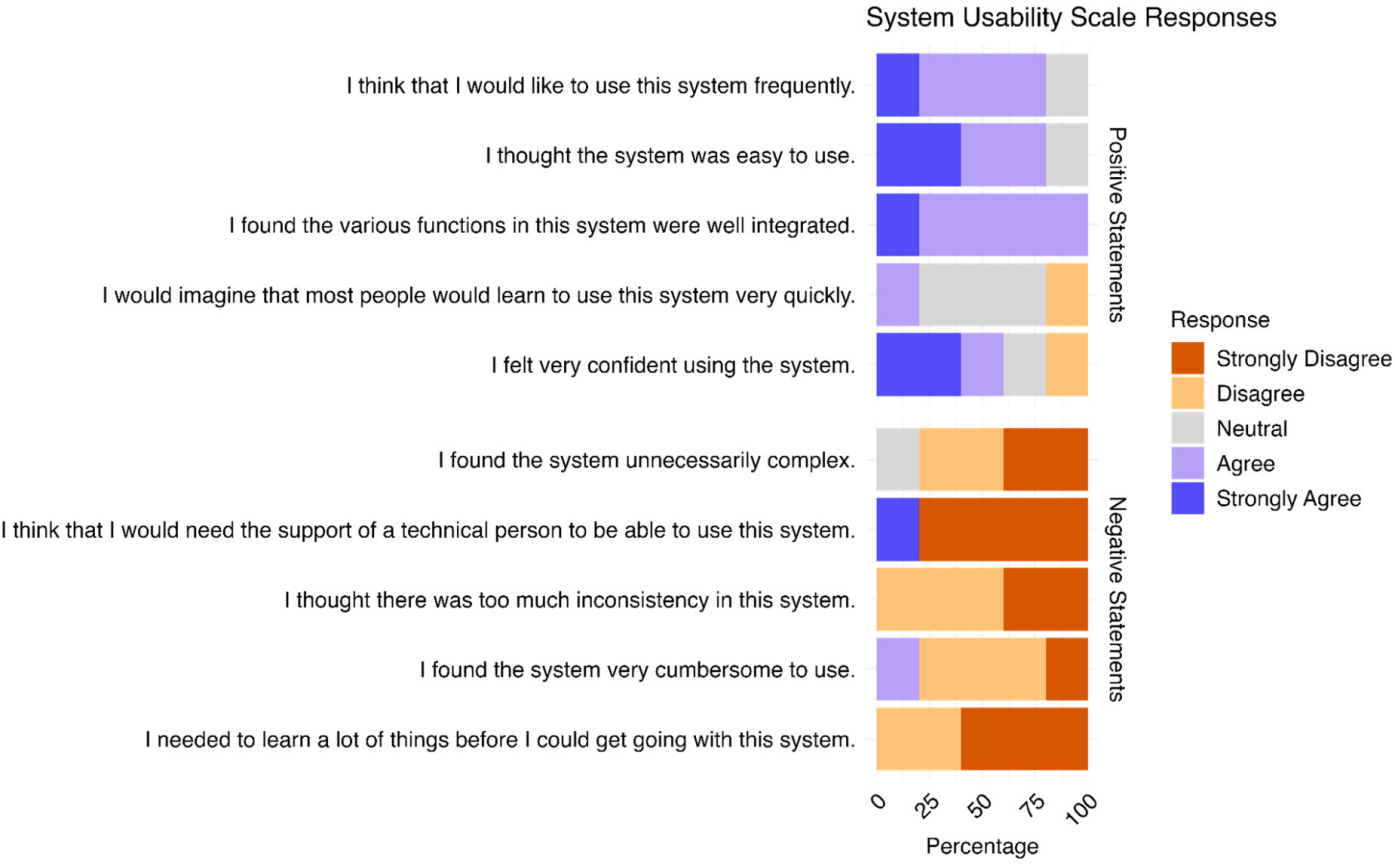
SUS of practitioners (*n* = 5).

SUS data were available for five out of the eight practitioners using Recovery Cat (see Figure 7, Supplemental Table S4**)**. The practitioners included two doctors in training, one specialist, and two consultant doctors, all in the field of psychiatry and working in the psychiatric outpatient department. The mean SUS score indicated good user-friendliness (76, sd = 15.87, SUS scores > 70 indicate good usability^31^). Most practitioners reported positive perceptions of the app, with 80% indicating they would like to use it frequently, 80% finding it easy to use, and 100% agreeing that its functions were well-integrated. However, unlike participants, most practitioners were neutral (60%) regarding the ease with which people could learn to use the system quickly. The majority of participants disagreed with negative usability aspects, such as the system being unnecessarily complex (80%), cumbersome to use (80%), or inconsistent (100%). Most (80%) reported not needing technical support to use the app, although one individual strongly agreed with the need for such support. All practitioners agreed that they did not need extensive training before using Recovery Cat.

SUS: System Usability Scale.

## Discussion

The present study investigated the feasibility and usability of a highly adaptable smartphone-based symptom monitoring system for patients with SMI. The results indicate that the monitoring system is feasible for use in this patient group, with 84.61% of participants completing at least half of the expected daily entries. The mean number of days between data entry was 1.48 (sd = 2.02). Usability ratings were high among patients (SUS = 82.4, sd = 11.57) and practitioners (SUS 76, sd = 15.87), respectively. These findings contribute to the growing body of evidence suggesting that smartphone-based RMBC applications could be a valuable addition to the treatment of patients with SMI and can be integrated into existing care pathways. They also demonstrate that clinicians can implement monitoring that is tailored to patient needs.

CGI and GAF indicated that the study participants suffered from SMI, not only in terms of their formal diagnosis but also regarding symptom severity and functioning. While the average adherence rate of 61.24% (55.5 out of 90 days) failed our expectations of at least 75% daily ePRO entries during the study period, findings showed that SMI patients engaged with the intervention at a moderate to high level. Specifically, the large group of participants using Recovery Cat almost every day (mean interval between data entries 1.48 days (sd = 2.02)), reflected a relatively frequent engagement. Furthermore, the finding that 84.61% (*n* = 22) exceeded 50% data entries and 96.15% (*n* = 25) exceeded 25% of data entries throughout their participation demonstrates the system's ability to continuously collect meaningful data for patients and clinicians.

From a clinical perspective, it is noteworthy that 84.61% of participants with SMI responded to at least 50% of the prompts. Given that data availability is typically limited between clinical encounters, this level of engagement represents a substantial improvement in continuous symptom monitoring. Importantly, Recovery Cat functioned without any additional human reminders,^32,33^ such as phone calls or text messages, which further illustrates its integrability in the outpatient care setting. The ability to collect patient-reported data at this frequency provides valuable insights that may enhance clinical decision-making and allow for more timely interventions.

While a study piloting EMA in psychiatric outpatients without any reminders or incentives for completion found that only 20% of the participants ever logged into the system,^34^ other studies investigating similar applications to Recovery Cat reported high acceptance and engagement rates.^35^

In the present study, participants with affective disorders tended to show higher adherence than those with schizophrenia or schizoaffective disorders. This aligns with prior results of a meta-analysis by Vachon et al. showing significantly lower retention rates in patients with psychotic disorders compared to depressive and bipolar disorders, which might be explained by the nature of symptoms, such as more severe cognitive or motivational impairments or even episodes of acute crisis.^19^ Adherence might also be influenced by both perceived therapeutic relevance and the therapeutic relationship between patients and health care practitioners. It has been shown that RMBC can enhance therapeutic alliance by empowering patients to be more informed and thus participate in shared decision-making.^36^,^37^. Future qualitative research should explore individual and contextual factors such as digital literacy, perceived burden of symptom tracking, motivation, and trust in symptom tracking, perceived integration into existing pathways or social support, and home environment.

We did not observe clear associations between demographic variables and engagement, nor did we find a significant relationship between the number of daily PROM items and adherence. However, in contrast to previous studies suggesting that an increasing number of ePROM items may increase patient burden and reduce compliance,^36,38^ we found a positive trend between an increasing number of ePROM items and the adherence rate. Our results may reflect the value of collaboratively selected and individualized ePRO items along with their review for RMBC. These findings align with previous work demonstrating that collaborative data review and shared decision-making enhance engagement in RMBC interventions.^39^ However, the adherence observed in our study also suggests room for improvement. Following a user-centered design methodology, we focused on rapid prototyping and iterative testing cycles.^40^ Consequently, the study was conducted at a relatively early stage of the development process, where technical issues might have led to nonadherence and to the high dropout rate.

The choice to focus on PROMs rather than passive sensing was guided by methodological considerations. Recovery Cat prioritizes active self-reporting, patient–provider collaboration, and patient empowerment. While passive sensing methods offer unique opportunities to capture physiological and behavioral parameters, they may present challenges for clinical interpretability when aiming to understand trajectories of SMI. Previous studies have shown that passive sensing via mobile devices can be acceptable to patients with SMI.^41^ Building on this, future studies with Recovery Cat may incorporate passive sensing components to complement ecological momentary assessments and provide a more comprehensive picture of SMI.

System usability was rated positively by patients (mean SUS = 82.4) and clinicians (mean SUS = 76). This suggests that Recovery Cat is intuitive and functional. Notably, the high ratings for ease of use indicate that users found the application accessible and not overly complex. Additionally, positive feedback regarding the quick learning process and the absence of the need for technical support suggests that the system can be implemented with minimal training, providing a scalable solution. The relatively lower practitioner SUS may either reflect challenges in perceived time investment or the lack of incentives of incorporating RMBC data into their outpatient sessions. In future versions of Recovery Cat, we plan to improve dashboard functionality through trend detection and redesign of the longitudinal data display.

### Limitations and future research directions

Several limitations should be acknowledged. First, the study was conducted with an MVP that may have introduced technical barriers, contributing to the 40.82% dropout rate. Technical issues limited overall usability and likely led to an underestimation of adherence. On the other hand, real-world adherence rates are usually lower as compared to research contexts. Recovery Cat may address this challenge through its emphasis on codesign, individualization and integration into routine care. Second, the study used convenience sampling, and participants likely had greater digital literacy and openness to trying app-based interventions. As a result, vulnerable subgroups, such as older adults or individuals with limited digital literacy or access to digital tools, may have been underrepresented. This limits generalizability and highlights the risk of further digital exclusion in real-world implementation. Individual causes for the lack of engagement remain elusive due to the limited availability of data on possible motivational deficits. To better understand user motivation, future research should incorporate qualitative methodologies such as interviews or focus groups. This would allow for deeper exploration on how users perceive the value, relevance, and usability of such tools. Fourth, in the current design, clinicians could access patient data during routine appointments and adjust the monitoring plan collaboratively. However, use of the dashboard varied, and technical usage logs were not systematically recorded due to a privacy-preserving design. Future studies should collect detailed clinician engagement data, including dashboard usage patterns and attitudes toward RMBC integration, to better understand how digital tools are used in real-world settings. Moreover, future studies should assess the clinical impact of highly adaptable smartphone-based monitoring to better establish the effectiveness of Recovery Cat. Finally, the next research phase should focus on evaluating long-term adherence and identifying factors that support or hinder sustained engagement over time. A more detailed exploration of the technical challenges and the implementation of continuous user support could help further improve the user experience and enhance usability.

## Conclusion

The results of this study suggest that a highly adaptable smartphone-based symptom monitoring is a feasible approach for patients with SMI, with promising adherence rates and positive usability feedback indicating the potential for this method to enhance mental health care in outpatient settings. Collaboration and joint decision-making in symptom monitoring empower patients to actively participate in their treatment, fostering engagement and self-awareness while ultimately strengthening the therapeutic alliance. By combining patient-reported data with clinical expertise, treatment can be dynamically tailored. This approach aims at enhancing patient satisfaction but can also facilitate early intervention, promoting recovery and long-term self-management. Despite the technical challenges and the need for further optimization of the user interface, the findings support the promise of this approach in improving patient engagement and enabling continuous symptom monitoring. Further research is needed to explore the long-term effectiveness and sustainability of this intervention and to address potential barriers to technical and organizational implementation, including suitable reimbursement schemes for implementing hybrid care and digital patient journeys.

## Supplemental Material

sj-docx-1-dhj-10.1177_20552076251393283 - Supplemental material for Highly adaptable smartphone-based monitoring for patients with severe mental illness: Feasibility and usability study Supplemental material, sj-docx-1-dhj-10.1177_20552076251393283 for Highly adaptable smartphone-based monitoring for patients with severe mental illness: Feasibility and usability study by Felix Machleid, Anette Schönewald, Esther Quinlivan, Linda Kokwaro, Louisa Schröder-Frekes, Toni Muffel, CasparWiegmann and Jakob Kaminski in DIGITAL HEALTH
