# Supplementary material for: Highly adaptable smartphone-based monitoring for patients with severe mental illness: Feasibility and usability study
Source: Digit Health. 2025 Nov 13;11:20552076251393283. doi: 10.1177/20552076251393283 (PMC12615936; doi:10.1177/20552076251393283)
Supplement: sj-docx-1-dhj-10.1177_20552076251393283 - Supplemental material for Highly adaptable smartphone-based monitoring for patients with severe mental illness: Feasibility and usability study [file sj-docx-1-dhj-10.1177_20552076251393283.docx]

**Supplementary Materials**

**Supplementary Table 1:** Additional Demographics and baseline characteristics of all participants, completers, and dropouts.

|  | **All partici-**  **pants (n)** | **Comple-**  **ters (n)** |
| --- | --- | --- |
| **Total** | 49 | 29 |
| **Marital status** |  |  |
| Not specified | 1 | 1 |
| Living with spouse | 9 | 5 |
| Living separately from spouse | 3 | 2 |
| Single | 31 | 18 |
| Divorced | 5 | 3 |
| Widowed | 0 | 0 |
| **Living conditions** |  |  |
| Not specified | 0 | 0 |
| Without a fixed residence | 1 | 0 |
| Apartment/house | 46 | 28 |
| Supported living | 1 | 1 |
| Retirement or nursing home | 0 | 0 |
| Other living facility | 1 | 0 |
| **Degree** |  |  |
| Not specified | 1 | 1 |
| No diploma | 0 | 0 |
| Secondary school / elementary school | 0 | 0 |
| Intermediate school /Technical college | 8 | 4 |
| Polytechnic secondary school | 0 | 0 |
| Matriculation | 4 | 2 |
| General higher education entrance | 35 | 22 |
| Other qualification | 1 | 0 |
| Still at school | 0 | 0 |
| **Employment** |  |  |
| Not specified | 0 | 0 |
| Full-time employment | 12 | 6 |
| Part-time employment | 12 | 8 |
| Partial retirement | 0 | 0 |
| Marginally employed (400€ job) | 0 | 0 |
| One-euro job | 0 | 0 |
| Irregularly employed | 0 | 0 |
| In vocational training/apprenticeship | 1 | 0 |
| In retraining | 0 | 0 |
| Volunteer service, voluntary social/ecological year | 0 | 0 |
| Maternity leave, parental leave, parental leave | 0 | 0 |
| Not employed (student, unemployed, pensioner) | 24 | 15 |
| ​​Retirement due to mental illness | 11 | 7 |
| **Occupation** |  |  |
| Not specified | 1 | 1 |
| Student at a secondary school | 0 | 0 |
| University student | 2 | 2 |
| Retired | 7 | 4 |
| Unemployed | 8 | 4 |
| Permanently unemployable | 4 | 3 |
| Stay at home | 1 | 1 |
| No category applies | 10 | 7 |
| Other | 3 | 1 |
| **Income** |  |  |
| Not specified | 1 | 1 |
| Under 1.000€ | 13 | 9 |
| 1.000 to 2.000€ | 8 | 4 |
| 2.000 to 3.000€ | 9 | 5 |
| >3000€ | 18 | 10 |

**Supplementary Table 2:** Adherence rates of individual participants

| **Record ID** | **Number of daily ePRO items** | **Number of individual daily ePRO items** | **Engagement duration in days** | **Days with ePRO entries** | **Adherence relative engagement (days with ePRO entries/days of engagement)** |
| --- | --- | --- | --- | --- | --- |
| 63 | 14 | 9 | 90 | 90 | 1.00 |
| 64 | 9 | 6 | 90 | 90 | 1.00 |
| 73 | 20 | 15 | 90 | 90 | 1.00 |
| 74 | 26 | 21 | 90 | 90 | 1.00 |
| 48 | 7 | 4 | 89 | 87 | 0.98 |
| 24 | 7 | 4 | 90 | 83 | 0.92 |
| 31 | 7 | 4 | 90 | 78 | 0.87 |
| 9 | 10 | 5 | 90 | 72 | 0.80 |
| 20 | 9 | 6 | 90 | 71 | 0.79 |
| 19 | 14 | 7 | 80 | 61 | 0.76 |
| 32 | 2 | 1 | 83 | 58 | 0.70 |
| 23 | 11 | 6 | 76 | 53 | 0.70 |
| 21 | 6 | 3 | 90 | 58 | 0.64 |
| 26 | 8 | 3 | 69 | 43 | 0.62 |
| 13 | 10 | 3 | 90 | 54 | 0.60 |
| 69 | 26 | 21 | 71 | 42 | 0.59 |
| 25 | 8 | 5 | 90 | 53 | 0.59 |
| 6 | 7 | 4 | 43 | 25 | 0.58 |
| 45 | 7 | 2 | 68 | 39 | 0.57 |
| 27 | 11 | 6 | 90 | 51 | 0.57 |
| 8 | 7 | 2 | 90 | 48 | 0.53 |
| 59 | 15 | 10 | 40 | 21 | 0.53 |
| 7 | 10 | 5 | 90 | 41 | 0.46 |
| 38 | 6 | 1 | 18 | 5 | 0.28 |
| 76 | 27 | 22 | 87 | 22 | 0.25 |
| 70 | 9 | 4 | 87 | 8 | 0.09 |

**Supplementary Table 3:** Exploratory subgroup analysis of engagement duration

|  | **Adherence in % (sd)** |
| --- | --- |
| **Gender** |  |
| Male (n = 18) | 67.30 (27.91) |
| Female (n = 7) | 65.81 (15.46) |
| Diverse (n = 1) | 69.88 (NA) |
| **Diagnosis** |  |
| Schizophrenia and Schizoaffective Disorders (n = 7) | 55.13 (15.10) |
| Affective Disorders (n = 19) | 71.37 (25.80) |
| F20: Paranoid Schizophrenia (n = 5) | 49.93 (13.82) |
| F25: Schizoaffective Disorder (n =2) | 68.12 (11.50) |
| F31: Bipolar Disorder (n = 10) | 64.92 (31.52) |
| F32/33: (Recurrent) Depressive Disorder (n = 9) | 78.54 (16.43) |
| **Degree** |  |
| Intermediate school / Technical college (n = 4) | 82.29 (20.37) |
| Matriculation (n =1) | 9.20 (NA) |
| General higher education entrance (n = 20) | 67.18 (21.91) |
| NA ( n =1) | 60.00 (NA) |
| **Income** |  |
| < 1.000€ (n =9) | 65.25 (20.97) |
| 1.000 to 2.000€ (n = 4) | 63.09 (30.92) |
| 2.000 to 3.000€ (n = 4) | 56.00 (37.11) |
| > 3000€ (n = 8) | 74.80 (20.53) |
| NA (n =1) | 80.00 (NA) |

**Abbreviations:** CGI = clinical global impression scale, n = number, NA = not applicable, sd = standard deviation

**Supplementary Table 4:** System Usability Scale items and breakdown of participants‘ and practitioners’ responses by percentage

| **SUS**  **item no** | **SUS item** | **Answer option** | **Partici-**  **pants (n)** | **Practitio-**  **ners (n)** |
| --- | --- | --- | --- | --- |
| 1 | I think that I would like to use this system frequently. | Strongly Disagree | 3 | 0 |
|  |  | Disagree | 2 | 0 |
|  |  | Neutral | 5 | 3 |
|  |  | Agree | 15 | 1 |
|  |  | Strongly Agree | 2 | 1 |
| 2 | I found the system unnecessarily complex. | Strongly Disagree | 17 | 2 |
|  |  | Disagree | 7 | 2 |
|  |  | Neutral | 2 | 1 |
|  |  | Agree | 0 | 0 |
|  |  | Strongly Agree | 1 | 0 |
| 3 | I thought the system was easy to use. | Strongly Disagree | 0 | 0 |
|  |  | Disagree | 0 | 0 |
|  |  | Neutral | 2 | 1 |
|  |  | Agree | 8 | 2 |
|  |  | Strongly Agree | 17 | 2 |
| 4 | I think that I would need the support of a technical person to be able to use this system | Strongly Disagree | 22 | 4 |
|  |  | Disagree | 1 | 1 |
|  |  | Neutral | 2 | 0 |
|  |  | Agree | 1 | 0 |
|  |  | Strongly Agree | 1 | 0 |
| 5 | I found the various functions in this system were well integrated. | Strongly Disagree | 1 | 0 |
|  |  | Disagree | 1 | 0 |
|  |  | Neutral | 7 | 0 |
|  |  | Agree | 14 | 4 |
|  |  | Strongly Agree | 4 | 1 |
| 6 | I thought there was too much inconsistency in this system. | Strongly Disagree | 12 | 2 |
|  |  | Disagree | 9 | 3 |
|  |  | Neutral | 4 | 0 |
|  |  | Agree | 1 | 0 |
|  |  | Strongly Agree | 1 | 0 |
| 7 | I would imagine that most people would learn to use this system very quickly. | Strongly Disagree | 0 | 0 |
|  |  | Disagree | 1 | 1 |
|  |  | Neutral | 2 | 3 |
|  |  | Agree | 10 | 1 |
|  |  | Strongly Agree | 14 | 0 |
| 8 | I found the system very cumbersome to use. | Strongly Disagree | 16 | 1 |
|  |  | Disagree | 10 | 3 |
|  |  | Neutral | 1 | 1 |
|  |  | Agree | 12 | 0 |
|  |  | Strongly Agree | 9 | 0 |
| 9 | I felt very confident using the system. | Strongly Disagree | 0 | 0 |
|  |  | Disagree | 0 | 0 |
|  |  | Neutral | 3 | 1 |
|  |  | Agree | 11 | 1 |
|  |  | Strongly Agree | 13 | 2 |
| 10 | I needed to learn a lot of things before I could get going with this system. | Strongly Disagree | 25 | 3 |
|  |  | Disagree | 2 | 2 |
|  |  | Neutral | 0 | 0 |
|  |  | Agree | 0 | 0 |
|  |  | Strongly Agree | 0 | 0 |

**Supplementary Table 5:** Mean and standard deviation (sd) of difference in days between data entry per participant.

| **Record ID** | **Mean** | **sd** |
| --- | --- | --- |
| 6 | 3.62 | 5.64 |
| 7 | 2.23 | 2.18 |
| 8 | 1.88 | 1.15 |
| 9 | 1.21 | 1.41 |
| 13 | 1.57 | 1.05 |
| 19 | 1.32 | 0.65 |
| 20 | 1.32 | 0.76 |
| 21 | 1.57 | 0.93 |
| 23 | 1.45 | 1.03 |
| 24 | 1.09 | 0.28 |
| 25 | 1.82 | 1.48 |
| 26 | 1.63 | 1.28 |
| 27 | 1.76 | 1.67 |
| 30 | 1.45 | 0.93 |
| 31 | 1.14 | 0.42 |
| 38 | 2.67 | 2.08 |
| 45 | 1.78 | 1.00 |
| 48 | 1.02 | 0.15 |
| 59 | 2.00 | 1.49 |
| 63 | 1.00 | 0.00 |
| 64 | 1.00 | 0.00 |
| 69 | 1.73 | 1.38 |
| 70 | 13.71 | 14.28 |
| 73 | 1.00 | 0.00 |
| 74 | 1.00 | 0.00 |
| 76 | 4.25 | 8.03 |
